# Supplementary material for: Dynamics of Lactic Acid Bacteria Dominance in Sour Bamboo Shoot Fermentation: Roles of Interspecies Interactions and Organic Acid Stress
Source: Foods. 2025 Oct 12;14(20):3481. doi: 10.3390/foods14203481 (PMC12563178; doi:10.3390/foods14203481)
Supplement: Supplementary file 1 [file foods-14-03481-s001.zip › foods-3907087-supplementary.pdf]

Supplementary material

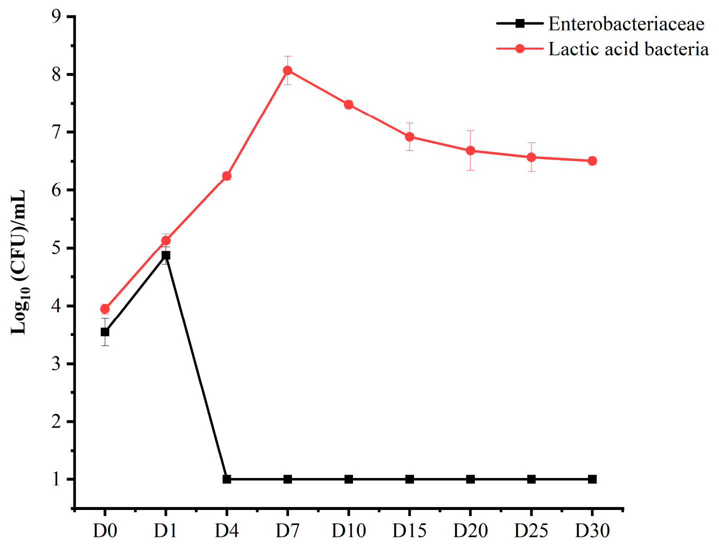

Figure S1. Plate count of *Enterobacteriaceae* and Lactic acid bacteria in sour bamboo shoots with different fermentation days. The horizontal axis represents time (days), and the vertical axis shows the log-transformed plate count data.

Table S1. Pearson correlation matrix of inter-genus relationships in sour bamboo shoot fermentation microbiota

r	Weissella	Lactiplantibacillus	Lactococcus	Limosilactobacillus	Raoultella	Lactobacillus	Escherichia-Shigella	Pantoea	Enterococcus	Streptococcus	Levilactobacillus	Klebsiella	Bifidobacterium	Acinetobacter	Ligilactobacillus	Brevundimonas
Weissella	1.00	-0.73	-0.43	-0.63	0.01	-0.80	-0.18	-0.10	0.91	-0.15	-0.66	-0.06	-0.15	-0.03	-0.16	-0.15
Lactiplantibacillus	-0.73	1.00	0.52	0.75	-0.58	0.87	-0.48	-0.52	-0.60	-0.50	0.79	-0.54	-0.49	-0.56	-0.49	-0.53
Lactococcus	-0.43	0.52	1.00	0.81	-0.70	0.45	-0.50	-0.63	-0.67	-0.50	0.36	-0.65	-0.52	-0.67	-0.48	-0.41
Limosilactobacillus	-0.63	0.75	0.81	1.00	-0.66	0.81	-0.50	-0.58	-0.68	-0.53	0.59	-0.62	-0.56	-0.63	-0.53	-0.49

Raoultella	0.01	-0.58	-0.70	-0.66	1.00	-0.40	0.96	0.99	0.10	0.97	-0.40	1.00	0.96	1.00	0.95	0.93
Lactobacillus	-0.80	0.87	0.45	0.81	-0.40	1.00	-0.28	-0.33	-0.61	-0.33	0.90	-0.37	-0.35	-0.38	-0.35	-0.36
Escherichia-Shigella	-0.18	-0.48	-0.50	-0.50	0.96	-0.28	1.00	0.98	-0.14	1.00	-0.28	0.98	0.99	0.97	0.99	0.99
Pantoea	-0.10	-0.52	-0.63	-0.58	0.99	-0.33	0.98	1.00	-0.02	0.99	-0.35	1.00	0.98	1.00	0.98	0.96
Enterococcus	0.91	-0.60	-0.67	-0.68	0.10	-0.61	-0.14	-0.02	1.00	-0.13	-0.42	0.02	-0.12	0.05	-0.16	-0.18
Streptococcus	-0.15	-0.50	-0.50	-0.53	0.97	-0.33	1.00	0.99	-0.13	1.00	-0.34	0.98	1.00	0.98	1.00	0.99
Levilactobacillus	-0.66	0.79	0.36	0.59	-0.40	0.90	-0.28	-0.35	-0.42	-0.34	1.00	-0.38	-0.36	-0.38	-0.37	-0.39
Klebsiella	-0.06	-0.54	-0.65	-0.62	1.00	-0.37	0.98	1.00	0.02	0.98	-0.38	1.00	0.98	1.00	0.97	0.95
Bifidobacterium	-0.15	-0.49	-0.52	-0.56	0.96	-0.35	0.99	0.98	-0.12	1.00	-0.36	0.98	1.00	0.97	1.00	0.99
Acinetobacter	-0.03	-0.56	-0.67	-0.63	1.00	-0.38	0.97	1.00	0.05	0.98	-0.38	1.00	0.97	1.00	0.97	0.94
Ligilactobacillus	-0.16	-0.49	-0.48	-0.53	0.95	-0.35	0.99	0.98	-0.16	1.00	-0.37	0.97	1.00	0.97	1.00	0.99
Brevundimonas	-0.15	-0.53	-0.41	-0.49	0.93	-0.36	0.99	0.96	-0.18	0.99	-0.39	0.95	0.99	0.94	0.99	1.00

Table S2. Correlation analysis between microbial genera and physicochemical parameters via coor.test

genus	env	r	p	rd	r.sign	pd
total sugar	Weissella	0.61	0.20	>= 0.3	Positive	P >= 0.05
reducing sugar	Weissella	0.53	0.28	>= 0.3	Positive	P >= 0.05
pH	Weissella	0.57	0.24	>= 0.3	Positive	P >= 0.05
total acid	Weissella	-0.64	0.17	>= 0.3	Negative	P >= 0.05
total sugar	Lactiplantibacillus	-0.82	0.05	>= 0.3	Negative	P < 0.05
reducing sugar	Lactiplantibacillus	-0.70	0.12	>= 0.3	Negative	P >= 0.05
pH	Lactiplantibacillus	-0.79	0.06	>= 0.3	Negative	P >= 0.05
total acid	Lactiplantibacillus	0.93	0.01	>= 0.3	Positive	P < 0.05
total sugar	Lactococcus	-0.49	0.32	>= 0.3	Negative	P >= 0.05
reducing sugar	Lactococcus	-0.35	0.49	>= 0.3	Negative	P >= 0.05
pH	Lactococcus	-0.93	0.01	>= 0.3	Negative	P < 0.05
total acid	Lactococcus	0.69	0.13	>= 0.3	Positive	P >= 0.05
total sugar	Limosilactobacillus	-0.73	0.10	>= 0.3	Negative	P >= 0.05
reducing sugar	Limosilactobacillus	-0.78	0.07	>= 0.3	Negative	P >= 0.05
pH	Limosilactobacillus	-0.89	0.02	>= 0.3	Negative	P < 0.05
total acid	Limosilactobacillus	0.87	0.02	>= 0.3	Positive	P < 0.05
total sugar	Raoultella	0.56	0.24	>= 0.3	Positive	P >= 0.05
reducing sugar	Raoultella	0.48	0.34	>= 0.3	Positive	P >= 0.05
pH	Raoultella	0.78	0.07	>= 0.3	Positive	P >= 0.05
total acid	Raoultella	-0.74	0.09	>= 0.3	Negative	P >= 0.05
total sugar	Lactobacillus	-0.93	0.01	>= 0.3	Negative	P < 0.05
reducing sugar	Lactobacillus	-0.92	0.01	>= 0.3	Negative	P < 0.05
pH	Lactobacillus	-0.65	0.17	>= 0.3	Negative	P >= 0.05
total acid	Lactobacillus	0.90	0.02	>= 0.3	Positive	P < 0.05
total sugar	Escherichia-Shigella	0.44	0.38	>= 0.3	Positive	P >= 0.05
reducing sugar	Escherichia-Shigella	0.41	0.42	>= 0.3	Positive	P >= 0.05
pH	Escherichia-Shigella	0.61	0.20	>= 0.3	Positive	P >= 0.05
total acid	Escherichia-Shigella	-0.61	0.20	>= 0.3	Negative	P >= 0.05
total sugar	Pantoea	0.51	0.30	>= 0.3	Positive	P >= 0.05
reducing sugar	Pantoea	0.44	0.38	>= 0.3	Positive	P >= 0.05
pH	Pantoea	0.70	0.12	>= 0.3	Positive	P >= 0.05
total acid	Pantoea	-0.68	0.14	>= 0.3	Negative	P >= 0.05
total sugar	Enterococcus	0.42	0.40	>= 0.3	Positive	P >= 0.05
reducing sugar	Enterococcus	0.33	0.53	>= 0.3	Positive	P >= 0.05
pH	Enterococcus	0.70	0.12	>= 0.3	Positive	P >= 0.05
total acid	Enterococcus	-0.56	0.25	>= 0.3	Negative	P >= 0.05
total sugar	Streptococcus	0.50	0.31	>= 0.3	Positive	P >= 0.05
reducing sugar	Streptococcus	0.47	0.34	>= 0.3	Positive	P >= 0.05
pH	Streptococcus	0.61	0.20	>= 0.3	Positive	P >= 0.05
total acid	Streptococcus	-0.64	0.17	>= 0.3	Negative	P >= 0.05

total sugar	Levilactobacillus	-0.97	0.00	>= 0.3	Negative	P < 0.05
reducing sugar	Levilactobacillus	-0.81	0.05	>= 0.3	Negative	P < 0.05
pH	Levilactobacillus	-0.53	0.28	>= 0.3	Negative	P >= 0.05
total acid	Levilactobacillus	0.85	0.03	>= 0.3	Positive	P < 0.05
total sugar	Klebsiella	0.54	0.27	>= 0.3	Positive	P >= 0.05
reducing sugar	Klebsiella	0.47	0.35	>= 0.3	Positive	P >= 0.05
pH	Klebsiella	0.73	0.10	>= 0.3	Positive	P >= 0.05
total acid	Klebsiella	-0.70	0.12	>= 0.3	Negative	P >= 0.05
total sugar	Bifidobacterium	0.53	0.28	>= 0.3	Positive	P >= 0.05
reducing sugar	Bifidobacterium	0.51	0.30	>= 0.3	Positive	P >= 0.05
pH	Bifidobacterium	0.61	0.19	>= 0.3	Positive	P >= 0.05
total acid	Bifidobacterium	-0.65	0.16	>= 0.3	Negative	P >= 0.05
total sugar	Acinetobacter	0.55	0.26	>= 0.3	Positive	P >= 0.05
reducing sugar	Acinetobacter	0.46	0.36	>= 0.3	Positive	P >= 0.05
pH	Acinetobacter	0.75	0.09	>= 0.3	Positive	P >= 0.05
total acid	Acinetobacter	-0.72	0.11	>= 0.3	Negative	P >= 0.05
total sugar	Ligilactobacillus	0.53	0.28	>= 0.3	Positive	P >= 0.05
reducing sugar	Ligilactobacillus	0.51	0.30	>= 0.3	Positive	P >= 0.05
pH	Ligilactobacillus	0.59	0.22	>= 0.3	Positive	P >= 0.05
total acid	Ligilactobacillus	-0.64	0.17	>= 0.3	Negative	P >= 0.05
total sugar	Brevundimonas	0.53	0.28	>= 0.3	Positive	P >= 0.05
reducing sugar	Brevundimonas	0.50	0.31	>= 0.3	Positive	P >= 0.05
pH	Brevundimonas	0.56	0.25	>= 0.3	Positive	P >= 0.05
total acid	Brevundimonas	-0.64	0.17	>= 0.3	Negative	P >= 0.05
